# Stability of Foams in Vacuum Drying Processes. Effects of Interactions between Sugars, Proteins, and Surfactants on Foam Stability and Dried Foam Properties

**DOI:** 10.3390/foods10081876

**Published:** 2021-08-13

**Authors:** Peter Kubbutat, Luísa Leitão, Ulrich Kulozik

**Affiliations:** Food and Bioprocess Engineering, TUM School of Life Sciences, Technical University of Munich, Weihenstephaner Berg 1, 85354 Freising, Germany; maria.luisa.leitao@gmail.com (L.L.); ulrich.kulozik@tum.de (U.K.)

**Keywords:** foam, saccharides, static light scattering, molecular interactions, surface tension, surface rheology, vacuum drying, microwave drying

## Abstract

The hypothesis was that saccharides mediate interactions between surface-active components and that this will have an impact on foam decay during the drying process. Static light scattering was performed to determine changes in interactions between the foam stabilizer on a molecular level. Furthermore, pendant drop and oscillating drop measurements were performed to examine the surface tension and surface rheology. Foams were dried in conventional dryers as well as microwave-supported vacuum dryers. Final foam properties were determined. It was shown that the addition of sugars, often added as protective substances for sensitive organic molecules, resulted in lower repulsion between different types of surface-active components, namely polysorbate 80 and β-lactoglobulin (β-lg). Differences in impact of the types of sugars and between different types of surfactant, protein, and small molecules were observed influencing the foam decay behavior. The interfacial properties of polysorbate 80 and β-lg were influenced by the type of the used sugars. The surface elasticity of protein stabilized surfaces was higher compared to that of polysorbate stabilized systems. Protein stabilized systems remained more stable compared to polysorbate systems, which was also affected by the used saccharide. Overall, a correlation between molecular interactions and foam decay behavior was found.

## 1. Introduction

In order to preserve sensitive, high-value food or pharmaceuticals components, products are stored refrigerated or even frozen. Another option is to dry products to prolong shelf life [1].

One way to remove water from products containing components sensitive to heat or oxidation is vacuum drying, which avoids excessive thermal stress by lower boiling point temperatures [2,3] as applied to oxygen-sensitive materials like fruits or proteins [4]. Sugars are often added as protective substances to prevent or mitigate structural changes and related losses in activity of organic material like enzymes or microorganisms. However, vacuum drying is limited in production capacity due to batch operation in most cases and the small size of vacuum chambers and long required drying times. Furthermore, the shrinkage of vacuum-dried products is higher compared to—e.g., freeze-drying—but lower than hot-air dried products [5,6]. Moreover, vacuum dried products are elastic and, therefore difficult to mill to obtain powders and they are slow in rehydration due to their compact structure.

One way to overcome most of these problems is to aerate the product before or at the beginning of the drying process [7,8]. Thereby, the surface area increases and the lamellae act as capillaries, transferring the water to the product’s surface, which results in faster drying [9] and a porous structure of the dried product [8,10,11]. However, the heat transfer into the product is lower, as the height of the product increases and the air voids inside the foam are acting as an insulator.

As the water transport occurs through the lamellae, the foam must be structurally stable, even drying processes imposing harsh conditions [7,12]. The main factors affecting liquid foam stability are disjoining pressure, viscoelasticity of the surfactant film, and interfacial tension. These factors are directly correlated with the leading causes of foam instability, namely liquid drainage, gas disproportionation, and bubble coalescence, all of which result in liquid film thinning and rupture [13,14]. Therefore, knowledge about the interactions between foaming agents, foam stabilizing additives, and foam stability under processing stress is of great importance, as these factors influence the foaming capacity of the starting solution and the resulting foam stability [15].

In order to improve the heat transfer into foams, microwave technology can be applied instead of heat conduction by heated shelves [16,17]. Microwaves are principally able to volumetrically heat the product across the entire product to the point of the moving drying front. As a result, the drying speed increases immensely [17]. Therefore, different researchers reported that the combination of foaming and vacuum drying is beneficial to increase the drying speed and to improve the structure of the dried product [12]. However, most of these studies are investigating protein stabilized and complex systems like fruits foams or bacteria suspensions [10,18,19]. In conjunction with added sugars, complex situations occur, which have not been intensely studied so far. Sugars might have an additional role in this regard, next to their protective effect against thermal and dehydration stress on sensitive organic molecules.

Few studies used small surfactants or stabilizing foaming agent-sugar combinations when studying foam stability during vacuum drying [20,21,22]. To the best of our knowledge, studies investigating the impact of surfactant-stabilized foams in accelerated microwave-assisted vacuum drying are lacking. Interactions of polysorbate 80 and various sugars, also added as protective agents of sensitive organic material like therapeutic proteins, enzymes, or microorganisms, and their impact on the foam properties was investigated in a previous study [23]. Therein, it was shown that sugars influence the interaction of surfactants at the air–water interface and change foam properties—mainly bubble size, firmness, or stability—depending on the used type of saccharide. However, surface rheology was not investigated, which is essential for the characterization of mechanical foam stability [15]. Hence, the impact of saccharides on different foam stabilizer systems regarding foam stability during vacuum and microwave-assisted vacuum drying is still largely unknown.

Therefore, the aim of the study was to investigate the influence of different sugar types and concentrations on the foam decay behavior during vacuum, and microwave-assisted vacuum drying of polysorbate and β-lactoglobulin (β-lg) stabilized foams. Sorbitol, maltose, sucrose, and maltodextrin were added to polysorbate 80 and β-lg solutions, and those complex solutions were investigated on a molecular, surface, and macroscopic level. Regarding the molecular interaction level, the second virial coefficient, A_2_, was determined to measure the impact of saccharide addition on intermolecular attraction or repulsion of surfactants or proteins as foaming agents. In order to be able to determine molecular interactions, β-lg was used instead of whey protein isolate. The results were used to explain differences in surface tension and surface rheology. Finally, it was aimed at finding a correlation between molecular interactions, interface properties, and dried foam properties. The hypothesis was that the sugars are interacting differently with proteins and surfactants due to H-bonds or conformational differences of the saccharide structure, which directly influence the interfacial properties and, therefore, the foam preservation in vacuum drying of foams. Furthermore, it was expected to gain relevant insights from comparing vacuum drying (VD) and microwave-assisted vacuum drying (MWVD) since different formulations will influence the heating properties during MWVD.

## 2. Materials and Methods

### 2.1. Materials

As foaming agent, polysorbate 80 (average molecular weight (M_w_) 1310 g·mol^−1^ [24], PS80, Sigma-Aldrich Chemie GmbH, Taufkirchen, Germany) and isolated β-lactoglobulin (M_W_ = 18.4 kg·mol^−1^, β-lg, in house production, purity >98%; method of production described in [25]) were used. The total protein content was measured with a VarioMaxCube (Elementar Analysensysteme GmbH, Langenselbold, Germany), which measures the nitrogen content in a sample with thermal combustion according to the method of Dumas [26]. The saccharides D-sorbitol (M_W_ = 182.2 g·mol^−1^, SOB, Gerbu Biotechnik GmbH, Heidelberg, Germany), D-maltose (M_W_ = 342.3 g·mol^−1^, MTO, Gerbu Biotechnik GmbH, Heidelberg, Germany), sucrose (M_W_ = 342.3 g·mol^−1^, Carl Roth GmbH, Karlsruhe, Germany), and maltodextrin DE-6 (M_W_ = 2880 g·mol^−1^ [27], MDX, Nutricia GmbH, Erlangen, Germany) were used as thickening agents. MilliQ-Water was used to prepare the solutions. Toluene was sourced from Merck (KGaA, Darmstadt, Germany).

Sample dispersions of 200 g each were prepared by mixing and dissolving 3% (*w*/*w*) β-lg or polysorbate 80 and different amounts of saccharide (i.e., 10%, 20%, 30%, 35%, or 40% (*w*/*w*)) in demineralized water. Sample solutions were dissolved at 20 °C and stirred at 200 rpm for 12 h in a 4 °C chamber to ensure complete dissolution of the added sugars. Before use, the samples were tempered at 20 °C in a water bath (F3, Fisher Scientific GmbH, Schwerte, Germany) and stirred using a magnetic stirrer (Maxi Direct, Fisher Scientific GmbH, Schwerte, Germany) at 200 rpm.

The critical micelle concentration (CMC) of PS80 in water was provided by the manufacturer with 13–15 mg∙L^−1^ or 0.012 mM at 20–25 °C [24].

### 2.2. Static Light Scattering Measurements

In order to determine the second virial coefficient A_2_, angular and concentration-dependent static light scattering measurements were performed using a ALV/CGS-3 goniometer system (ALV-Laser Vertriebsgesellschaft mbH, Langen, Germany). The goniometer was equipped with a 22 mW HeNe laser, providing coherent and monochromatic light with a wavelength of 632.8 nm at 20 °C. The experimental design was based on that of Antipova et al. [28], which were using static light scattering at for the investigation of surface activity of 11S globulin. For the measurement, angles between 30° < θ < 150° were utilized with 10° increments. Three consecutive individual runs with a data collection time of 10 s were averaged for each angle observation. Data was rejected, and runs were repeated if the intensity fluctuation was higher than 10%. The refractive indices *n* of all solvents and toluene were measured using the refractometer 74268 (Carl Zeiss AG, Oberkochen, Germany).

Samples with 1% (*w*/*w*) β-lg or 3% (*w*/*w*) polysorbate 80 in combination with different maltodextrin (0–2.5% (*w*/*w*)), maltose, sucrose, and sorbitol (1–7.5% (*w*/*w*)) concentrations, regarded as solvents, were investigated against toluene. For data analysis (i.e., static Berry plot analysis [29]), the ALV Correlator Software Version 3.0 (ALV-Laser Vertriebsgesellschaft mbH, Langen, Germany) was used to determine the second osmotic virial coefficient A_2_. Thereby, extrapolation of the scattering vector q as well as the protein concentration c towards zero was done by applying a linear regression model, respectively, according to the method also described by Dombrowski et al. [30].

### 2.3. Pendent Drop and Oscillating Drop Measurement

Surface tension and surface rheology of polysorbate 80 (3% *w*/*w*), as well as β-lactoglobulin (3% *w*/*w*) at different concentrations of sorbitol, maltose, sucrose and maltodextrin (0–40% *w*/*w*), were measured using a DSA100 (Krüss GmbH, Hamburg, Germany) at 20 °C. For the analysis, the samples were transferred into a syringe, and a drop of 12 µL was pressed out of a stainless-steel needle with a diameter of 1.81 mm. The ADVANCE software (Krüss GmbH, Hamburg, Germany) was used for data analysis and processing, which allowed the determination in the water-air phase of drop surface tension and rheology.

For pendent drop measurement, the picture analysis was done for the first 30 s with 10 frames per second (fps), thereafter for 1000 s with 2.5 fps and finally for 6170 s with 0.25 fps. Therefore, the evaluation of surface tension was monitored for a duration of 7200 s in total for each drop. The surface tension was computed by the software using Young–Laplace formula. The initial slope of surface tension over time (e.g., dσ/dt = 10 s) was used to determine the surface activity according to the description of Marinova et al. [31].

For the determination of the surface rheology, the surface of the formed drop firstly was brought into quasi-equilibrium, which for polysorbate samples was after 30 min and for β-lg samples was 60 min. Thereafter, the drop was subjected to a sinusoidal oscillation for 100 s with a frequency of 0.1 Hz at an amplitude of 400‰. The oscillatory deformation of the drop surface was ~3.2–5%, which was proved to be in the linear viscoelastic region (data not shown). Three consecutive individual runs were performed for each drop. The complex viscoelastic (E), elastic (storage modulus, E’), and viscous (loss modulus, E”) moduli were calculated by the software using data fitting according to Lucassen and Van den Tempel [32]. The tan (φ) was calculated according to Conde and Rodriguez Patino [33]

(1) $$\tan\;\left(\varphi \right)=\frac{E^{\prime \prime }}{E^{\prime }}$$

### 2.4. Foam Formation and Vacuum Drying

In order to produce foams under comparable conditions, 200 g solutions were prepared for each sample. After sample preparation as described in 2.1, 150 g of solution was stirred with a planetary rotor–stator mixer (KitchenAid ARTISAN 5KSM150PS, Whirlpool Corp., Greenville, Ohio, USA) with a wire wisp geometry at 220 rpm and 20 °C for 15 min. Directly after that, 15 g of foam was gently transferred into a cylindrical crystallization glass with d = 200 mm (VWR International GmbH, Darmstadt, Germany). Afterwards, the sample was immediately transferred into the drying plant.

For conventional vacuum drying, a freeze-dryer model Delta 1-24LSC (Martin Christ Gefriertrocknungsanlagen GmbH, Osterode am Harz, Germany) was used. The shelf temperature was set to 20 °C and the pressure was reduced to 15 mbar. The foams were dried for 16 h, except for the foams that completely collapsed. For the latter, the drying process was stopped 30 min after the collapse occurred because no further re-formation of foamy structures was expected 30 min after foam collapse.

The microwave-supported vacuum drying processes were performed using a microwave dryer model µVac0150fd (Püschner Microwave Power Systems GmbH & Co. KG, Schwanewede, Germany). The microwave drying plant was controlled by the software µWaveCAT (Püschner Microwave Power Systems GmbH & Co.KG, Schwanewede, Germany) and allowed process monitoring by the measured weight, temperature, or pressure. The sample weight was measured by an inline scale, which was connected to the turntable, and the product temperature was assessed by a pyrometer. A detailed description of the drying plant can be found in Ambros et al. [16]. The drying process was performed at 15 mbar, 80 W microwave power input, and 20 °C max. product temperature. The process was stopped after no mass loss occurred during 10 consecutive minutes or when the foam collapsed during the drying process.

At least two dryings for each formulation were performed.

### 2.5. Analysis of Dried Samples

Samples, which did not collapse during the drying process were characterized by the residual water content and water activity. In addition, the shape of the solidified pores and the overall foam structure were investigated. For the evaluation of water content and water activity, the dried samples were ground inside an electric coffee mill (PC-KSW 1021, Mühle Clatronic International GmbH, Kempen, Germany) for 15 s in order to obtain a homogeneous powder.

The residual water content was measured using Karl-Fischer titration. The analysis was conducted using a volumetric compact titrator V20S (Mettler-Toledo GmbH, Gießen, Germany). After a pre-titration period of 300 s, about 0.05 g sample was added and dissolved for 300 s in a 1:1 (*v*/*v*) mixture of Hydranal^®^-Formamid (Fisher Scientific GmbH, Schwerte, Germany) dry and Hydranal^®^-Methanol Rapid (Fisher Scientific GmbH, Schwerte, Germany). After dissolution, the titration with iodine started immediately. A two-fold determination of the moisture content was done, which, considering duplicate dried foams, resulted in a four-fold determination of moisture content.

The water activity was conducted in a HygroLab (Rotronic AG, Bassersdorf, Germany) at 25 °C. The measurement was stopped automatically after the value was in equilibrium. A two-fold determination of the water activity was done, which considering duplicate dried foams, resulted in a four-fold determination of the water activity.

### 2.6. Statistical Analysis

Error bars represent the standard deviation of samples. Lines are guide to the eye. Statistical significance of mean values was evaluated using OriginPro software (Originlab Corp., Northampton, MA, USA).

## 3. Results and Discussion

### 3.1. Influence of the Type of Sugar on the Interactions between Surface-Active Components

In order to assess the molecular interactions and behavior of the different foaming agent–sugar systems, A_2_ was obtained through the employment of the Berry’s method [29] to measured SLS data. Briefly, the A_2_ value indicates the direction and magnitude of overall intermolecular forces between two particles in solution by its charge and value, respectively. Positive A_2_ values correspond to net repulsive forces (where protein–solvent interactions are favored over protein–protein interactions), whereas negative values represent net attractive forces [34].

The second virial coefficient, A_2_, of polysorbate and β-lactoglobulin in the presence of sorbitol, sucrose, maltose, and maltodextrin is shown in Table 1. The values indicate a trend of repulsive or attractive interactions due to the addition of the investigated saccharides at high sugar concentrations.

For polysorbate samples, the interactions are between 1.38·10^−7^ for sorbitol and 1.08·10^−7^ mol·dm^3^·g^−2^ for sucrose, respectively. For β-lg, the value for sucrose (14.2·10^−7^ mol·dm^3^·g^−2^) was the highest, while maltose showed the lowest value (6.8·10^−7^ mol·dm^3^·g^−2^). In other words, the values were much lower for polysorbate samples compared to β-lg samples. However, all samples showed positive A_2_ values, thus indicating that the molecules of the foaming agent repulsed each other in the presence of saccharides and that the tendency for aggregation was lowered as well.

Since for polysorbate samples, the used concentration was much higher than the CMC [24], it can be assumed that the molecules were associated with forming spherical micelles [35,36]. As PS80 is a non-ionic surfactant and the sugars are neutrally charged, electrostatic interactions between polysorbate and saccharides were not likely to play a role. This would also be an explanation for the differences in A_2_ between PS80 and β-lg samples. Due to the lack of electrostatic interactions, H-bonds must have been the major interaction type, as already discussed in a previous study about the foaming properties of PS80 foams in the presence of saccharides [23]. However, effects like molecular crowding or the incorporation of PS80 into maltodextrin were not observed in the obtained data. This might be due to concentration differences between the previous (up to 60% saccharide concentration) and the actual study (up to 7.5%).

For all investigated saccharides, the A_2_ values were 1.3 ± 0.1·10^−7^ mol·dm^3^·g^−2^. This is remarkably similar to the A_2_ values, found by Richtering et al. [37] for octa(ethylene glycol) n-tetradecylether (C_14_E_8_) in water at 20 °C (1.1·10^−7^ mol·dm^3^·g^−2^). No significant differences between sugar types were detected. This was unexpected, as the investigated sugar types were assumed to have a different ability to form H-bonds due to a different number of potential H-bonding groups and structural differences. Comparable effects were also described by Ali et al. [38] during their investigation of different types of sugars in aqueous glycine solutions. Furthermore, the modification of hydrophobic interactions between non-ionic surfactants resulting from added saccharides as described by Claesson et al. [39] was not detectable. Therefore, it appears likely that the concentration of polysorbate was too high or the concentration of saccharides was too low to detect differences.

In comparison, differences between sugar types in protein samples were more pronounced. All β-lg samples showed a high positive A_2_ indicating a higher repulsive character than in the samples containing PS80. One explanation for the high virial coefficient would be that β-lg has a highly negative zeta-potential at neutral pH [40] resulting in strong repulsive forces between single molecules. However, the obtained A_2_ values were lower than the reported A_2_ value for β-lg in pure water by Dombrowski et al. [30], which was about 15·10^−7^ mol·dm^3^·g^−2^. Hence, it seems that the saccharides shielded the surface charge and lowered the repulsive forces down to nearly a third of the value of that of no added saccharide. This could be explained by the preferential exclusion mechanism as reported by Timasheff et al. [41,42]. Due to the higher affinity of the used saccharides for water compared to the affinity of β-lg, water migrated from the protein surface to the bulk solution, leaving the protein preferentially hydrated. Furthermore, the saccharide, now occupying layers adjacent to the hydration layer that surrounds the protein, can act as shielding matter. Thereby, it reduced the charge interactions between β-lg molecules and excluded volume between the proteins in the solution. In total, the repulsive character decreased with the increasing ability of preferential exclusion. Comparable observations were also made by Antipova et al. [28] during their investigation of the influence of dextran and maltodextrin on the surface activity of 11S globulin at the air–water interface. Furthermore, maltodextrin could also sterically hinder interactions between protein molecules due to its molecular size and potentially form clusters, resulting in an effective decrease of A_2_ towards zero. Nonetheless, it should be mentioned that maltodextrin consists of saccharides with different chain lengths [43,44,45]. Therefore, the data correspond to a saccharide mixture, which might influence the value obtained.

Therefore, differences between the sugar types can be attributed to the different structural and chemical properties of the saccharides. Various sugars have different abilities to form H-bonds [46,47] and/or to sterically disturb interactions between proteins due to structural differences [38].

### 3.2. Impact of Sugar Type and Concentration on the Surface Tension and Surface Activity of Polysorbate and β-Lactoglobulin Stabilized Foams

Besides the second virial coefficient, the surface tension and the surface activity were determined. It was expected that the observed differences in higher A_2_ influence the surface activity of foaming agents as also observed for 11S globulin in the presence of different saccharides [28]. Surface tension values of sugar dispersions without surfactant are published in Kubbutat & Kulozik [23] and showed only a slight decrease in surface tension with increasing saccharide concentration.

**Figure 1:**
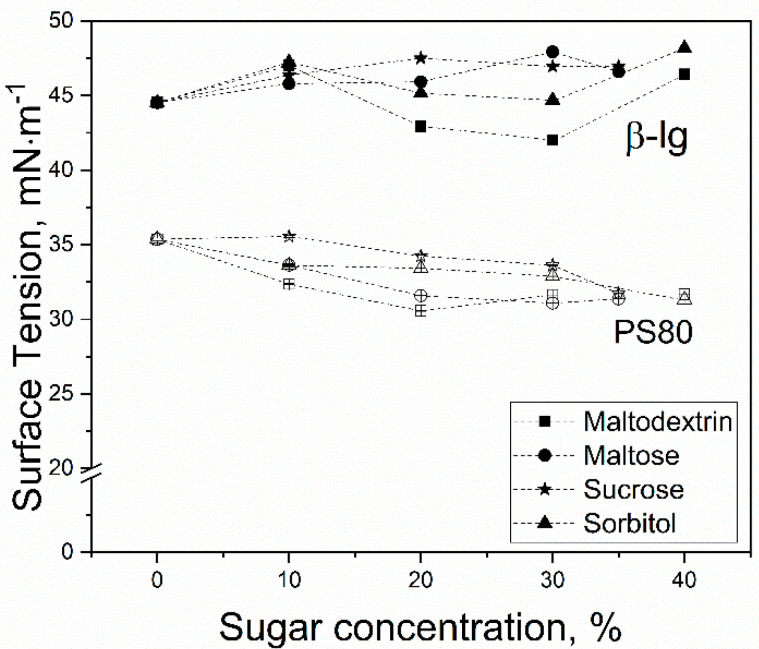
Surface tension of polysorbate 80 (empty symbols) and β-lactoglobulin (filled symbols) with different concentrations of added sugar after an equilibrium time of 7200 s at 20 °C, where (■) corresponds to maltodextrin, (●) to maltose, (★) to sucrose, and (▲) to sorbitol, respectively.

The impact of sugar concentration on the surface tension is shown in Table 2 and Figure 1. It is clearly observable that the final surface tension of β-lg samples was almost independent of the used sugar and higher compared to polysorbate samples. Furthermore, with the addition of saccharides, β-lg showed slightly higher surface tensions than the control. In contrast, for polysorbate samples, it was found that the higher the saccharide content, the lower the surface tension.

With regard to Section 3.1 polysorbate 80 has a lower A_2_ value compared to β-lg. Thereby, the polysorbate molecules do not repel as strongly as proteins, and the resulting concentration at the surface is higher. In addition, all sugars seem to moderate the interaction between polysorbate 80 and air, as the surface tension lowers with saccharide concentration. This is in accordance with a previous study on the influence of sugars on the foam properties of polysorbate 80 foams [23] and can be explained by strong hydrogen bonds between sugar and polysorbate as well as between sugar and water. Furthermore, Staples et al. [48] stated that sorbitol is able to interact with the ethylene-oxide groups of polysorbate and adsorb at its hydrophilic head group, resulting in lower surface tension. The formation of H-bonds can also be observed by the decrease of CMC of non-ionic surfactants in the presence of saccharides, as found by Acharya et al. [49]. The authors stated that the reduction in CMC was a result of water–carbohydrate interactions, which were lowering the monomer-stabilizing so-called ‘iceberg formation’ around non-polar tails of surfactants [50]. The ‘iceberg formation’ became less pronounced through the strong H-bonds between water and sugar, resulting in the destabilization of the surfactants monomer state followed by a faster formation of micelles and, therefore, a lower CMC [49]. As this effect seemed to appear for all investigated saccharides, the surface tension lowered with increasing sugar content.

The high repulsion can explain the higher surface tension of protein samples between the protein molecules described and discussed in Section 3.1. As shown in Figure 1, there was no constant trend for surface tension in dependency of the sugar concentration observable. However, as A_2_ decreased due to the addition of sugar, the surface tension should do so, too, like molecules at the air–water interface repelled each other less. However, this was not observed. A possible explanation might be that the A_2_ value was obtained from different sugar concentrations, and therefore, an overall trend was described. This might also be the reason for the high standard deviation of A_2_.

However, the increasing surface tension might also be a result of two contradictive mechanisms. On one side, the sugars are shielding the charges of the proteins resulting in higher surface concentration, but on the other side, they are stabilizing the natural conformation of the proteins, which made the protein interaction with the surface unfavorable. Antipova et al. [28] found during their study of 11S globulin in the presence of sucrose and glucose that proteins have an increased affinity to the aqueous phase with added saccharides and contributed this to strong hydrogen bonds resulting in excluding volume. Even though the concentrations used in our study were much higher, we also detected concentration-dependent changes. One explanation for this could be solvophobic effects, which might have occurred due to strong hydrogen bonds between sugar and water. Thus, the water is less accessible for the protein, whereby the proteins gain an increased affinity to move to the hydrophobic air bubble surface. Nevertheless, even though the saccharide concentration was higher than in the study of Antipova et al. [28], the mechanisms leading to the shift of surface tension seem to be comparable. An increasing surface tension was also found for the addition of erythritol or sucrose to WPI dispersions in studies of Nastaj et al. [51,52]. The increasing surface tension was explained by less adsorption of proteins at the air–water interface due to the high viscosity of the bulk phase [52] or differences in protein concentrations [51].

The surface activity, calculated by the method of Marinova et al. [31], is shown in Table 2. For β-lg samples, it can be observed that the surface activity for sucrose and maltodextrin was increasing with increasing saccharide concentration, while for sorbitol and maltose, the value was slightly decreasing. Polysorbate samples were showing stable or slightly increasing surface activities up to a saccharide concentration of 30%. Only for maltodextrin-PS80 samples, the surface activity was steadily decreasing. If the surface activity had an influence on the foam decay during the drying process, it would be expected that for samples with higher surface activity, the foams remain most stable. The reason is that the foam expands during the drying due to evaporation, heating, and low pressure, whereby the surface area increases, which has to be newly covered by foaming agents.

The A_2_ values for all PS80-carbohydrate mixtures were similar, which was unexpected. However, the explanation might be that the carbohydrates moderated the interactions of PS80 via their H-bonds and the formation of clusters, as discussed above. Thereby, the surface activity increased. At high sugar concentrations, the viscosity of the samples increased so much that the mobility of the PS80 molecules was decreased, resulting in slower surface adsorption. In addition, the formation of sugar clusters, which could integrate PS80, would lower the surface activity [53]. Consequently, at a particular saccharide concentration (here, 30 wt %), the surface activity would significantly decrease.

For maltodextrin, a different behavior was observed as the surface activity decreased steadily. An explanation for this might be that maltodextrin can interact and form complexes with surfactants, as reported by several researchers [54,55]. Semenova et al. [54] reported that maltodextrin was able to integrate PS80 into the structure of MDX due to H-bonds between the polyethylene-head of PS80 and the OH-groups of MDX. In addition, Wangsakan et al. [55] explained that complexes between MDX and surfactants might have a different impact on the surface tension depending on the surface-active component resulting from the promotion of the formation of 3D maltodextrin structures. Furthermore, maltodextrin might be able to sterically hinder the surfactant from moving to the surface [53], which should also be dependent on the size and structure of the foaming agent. This might also be an explanation for the detection of a different trend for β-lg compared to PS80.

The increasing surface activity of β-lg samples can be explained by the excluded volume, as described above [28]. However, sorbitol and maltose showed decreasing trends. As carbohydrates can form H-bonds in different numbers and strengths [38], it seems logical that the carbohydrates differ in their behavior. The high ability to moderate hydrophobic interactions of sorbitol might explain the observed decreasing surface activity. As sorbitol stabilized the native structure of the proteins [56], this effect might be strengthened by higher concentration, and the affinity of β-lg to the aqueous phase might be higher, thus decreasing the surface activity [28].

In summary, a correlation between the second virial coefficient and the surface tension was identified. However, differences in carbohydrates do not seem to be directly in accordance with this, which can be attributed to a too-generous statement and experimental design of the A_2_. In order to gain more detailed information about the influence of molecular interactions between the foaming agents and sugars, it would be helpful to study concentration-dependent values for A_2_ additionally. Regarding the previously discussed data, it was expected that the surface rheology would be significantly influenced by the sugars, as their presence affects the surface activity of the foaming agents [57].

### 3.3. Impact of the Sugar Type and Concentration on the Surface Rheology of Polysorbate and β-Lactoglobulin Stabilized Foams

**Figure 2:**
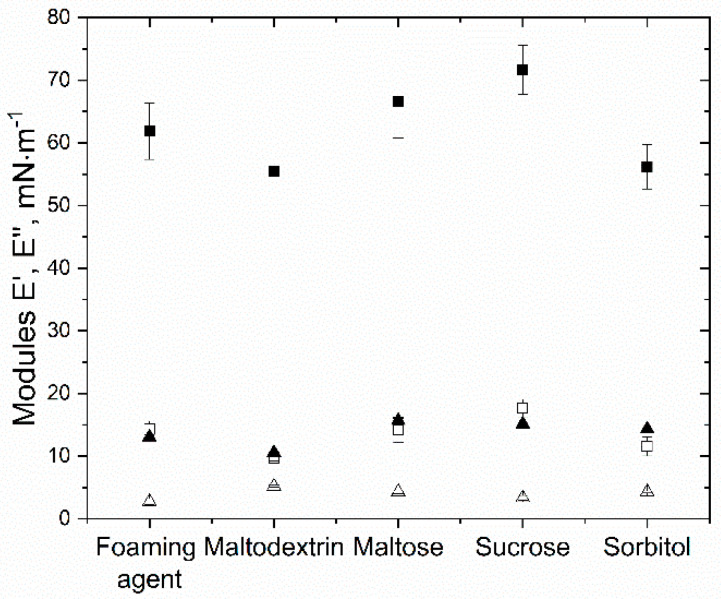
Influence of sugar type on the elastic (E’, squares) and viscoelastic (E’’, triangles) moduli of polysorbate (empty symbols) and β-lactoglobulin (filled symbols) samples pure or with a saccharide concentration of 20%.

The influence of sugar type on the surface rheology is shown in Figure 2. It can be clearly observed that the elastic moduli of polysorbate 80 samples were much lower than of protein systems. Furthermore, apparent differences between the investigated sugar types were detectable. The elastic modulus E’ of 3% β-lg without saccharide was around 60 mN·m^−1^, which seems comparable to the obtained E’ values reported by Lexis and Willenbacher [58] for 1% β-lg solutions. The high elastic moduli of protein samples can be explained by a structural rearrangement of proteins at the air–water interface. Thereby, proteins were interacting with each other via hydrophobic interactions or aggregation and ionic interactions at the hydrophilic parts of the proteins, which prevent detachment from the surface. The added saccharides promoted those interactions by H-bond formation in the presence of saccharides. Thereby, the proteins at the surface connect with each other, and a high viscoelastic behavior can be observed, as shown by other research groups [30,40,59]. In addition, the increase of E’ due to the addition of saccharides can be attributed to preferential exclusion, as discussed in the previous sections. Furthermore, due to lowering repulsive forces (see Section 3.1), the interactions between proteins are stronger, resulting in a more elastic behavior, as also was observed and described for the addition of ions by Dombrowski et al. [30].

As already discussed, the major interactions between saccharides and polysorbate are H-bonds. As the interactions between polysorbate molecules at the interface were expected to be much lower when compared to those of proteins, it was assumed that they would easily detach and attach at the surface, as well as move along the surface layer to compensate gaps of lack of surfactant in the interface [13,60]. In the presence of saccharides, this would be even easier, as the CMC is decreasing with increasing sugar content [48,61]. However, for disaccharides, only a small increase in E’ was observable.

**Figure 3:**
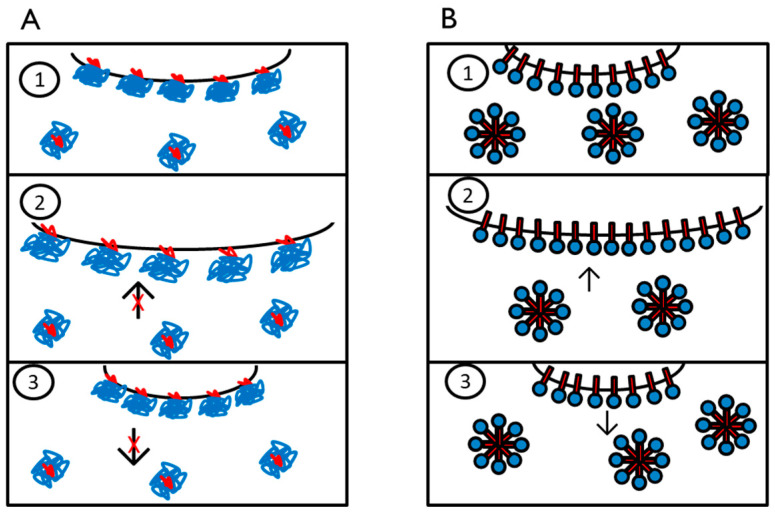
Schematic differences at the air–water interface between β-lactoglobulin (**A**) and polysorbate 80 (**B**) samples during oscillation.

In Table 3, the calculated tan (φ) for samples with 20% saccharide content is shown. A tan (φ) of zero would represent a perfect elastic behavior of the film [33,62]. The highest difference between the protein and surfactant samples was detected for maltodextrin (β-lg = 0.18 ± 0.01, PS80 = 0.54 ± 0.01). For maltose and sorbitol, surfactant samples had about a 40–50% higher tan (φ) value, which is following the already discussed behavior of PS80 at the surface. For 20% sucrose, no clear difference was obtained in comparison to the foaming agent. This was attributed to the low concentration since tan (φ) was influenced at higher sucrose concentrations. A much lower tan (φ) was also observed by Davis et al. [15] during their investigation of the impact of sucrose on the rheological properties of different proteins. However, Davis et al. [15] did not find a specific explanation but highlighted the importance of knowledge about the properties of the solutions to understand the interfaces’ rheological properties. Nonetheless, with increasing concentration, sucrose followed a clear trend as visibly observable in Figure 3. Therefore, it was postulated that the change in tan (φ) required a higher concentration than 20%.

**Figure 4:**
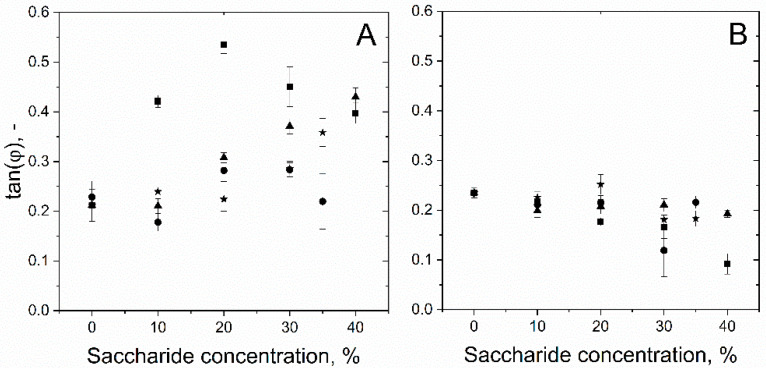
Influence of different saccharide concentrations on the calculated tan (φ) of polysorbate 80 (**A**) and β-lactoglobulin (**B**) samples at the air–water interface, with sorbitol (▲), sucrose (★), maltose (●), and maltodextrin (■).

The influence of sugar concentration on the tan (φ) will be discussed, considering the different foaming agents. Samples with polysorbate are shown in Figure 4A. It was clearly observable that with increasing saccharide concentration, the tan (φ) also increased. Therefore, it was assumed that this was linked to the higher viscosity of the bulk phase near the interface.

As we think that polysorbate had a high ability to attach and detach from the surface (Figure 3), the reattachment during oscillation might have been hindered due to the high viscosity of the solution or by steric hindrance of saccharide molecules and H-bonds between polysorbate and saccharides. Thereby, the surface became more brittle, as the newly formed surface during expansion was not immediately covered by polysorbate and the tan (φ) consequently increased. This would also agree with the obtained data of surface activity, which decreased sharply as discussed in Section 3.2. One exception was again maltodextrin, which had the most significant changes within the investigated range of saccharide concentration and decreased clearly between a concentration of 20% and 40%. While the increase of tan (φ) can also be explained by the increase of viscosity and the hindrance of reattachment of the surfactant, the decrease of tan (φ) must have a different origin. As this behavior only occurred with maltodextrin and polysorbate, it was assumed that the interactions between polysorbate and maltodextrin must be responsible for that behavior. Since maltodextrin can incorporate polysorbate molecules [54], those complexes might be located around the interface. Thereby, the MDX integrated polysorbate molecules could stabilize the surface during oscillation as MDX decreases their mobility, and thus, the detachment process would be less likely. However, this effect can only appear if other MDX molecules hinder the MDX-PS80 complexes from moving, as it is assumed to occur at high polysaccharide concentrations [53].

**Figure 5:**
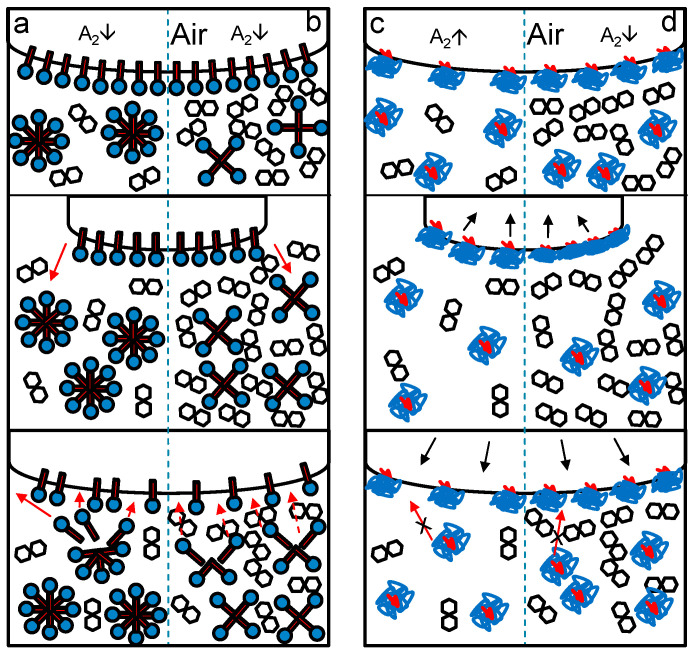
Schematic comparison of the impact of low (**a**,**c**) and high (**b**,**d**) sugar concentrations on the behavior of surfactants (**a**,**b**) and proteins (**c**,**d**) at the air–water interface.

In contrast, samples with β-lg showed decreasing tan (φ) with increasing sugar concentration. This can be attributed to the stabilization of the surface by the formation of H-bonds that moderate the interactions between proteins. Due to the addition of sugars, A_2_ decreased, promoting the intermolecular interactions between proteins. Furthermore, the sugars can be preferentially excluded and hence can form a shell around the protein layer at the surface. Thereby, the surface stability and consequently elasticity increased with increasing sugar concentration. Therefore, the observed behavior was in accordance with the obtained results from SLS and PDM. A scheme of how the authors imagine that sugar concentration influenced the surface rheology is shown in Figure 5.

Taking Section 3.1, Section 3.2 and Section 3.3 into consideration, it was expected that foams stabilized with β-lg would show less decay over time than those stabilized with polysorbate. The reason for this statement is the increasing elastic behavior of the surface with added sugar and the increased stability of the interface due to H-bonding between sugars and proteins. Since the surface activity of surfactant-saccharide formulations decreased sharply with high sugar content, it was expected that the advantage of small surfactants (e.g., the fast adsorption at the interface) would be neglected, resulting in a higher chance of foam collapse.

### 3.4. Drying Behavior of with Sugar Thickened Foam Matrices

On the basis of the observed differences in molecular interactions and surface properties of different saccharide-foaming agent mixtures as discussed above, their influence on the structural stabilization of foams during vacuum drying, different vacuum drying experiments were assessed to correlate sample properties and drying results. As the molecular interactions and surface rheology discussed in the previous sections are still present within the different formulations, but sample and not process-related, the discussion about the aforementioned will be brief. Nonetheless, the relevant sample properties show great influence, as shown below.

The residual water content (10 ± 2%) and the water activity (0.35 ± 0.1) for all investigated samples were independent of the used formulation. At the same time, the drying time for CVD was constantly set to 16 h, and the MWVD needed between 1.3 and 2 h until the drying was finished (data not shown).

**Figure 6:**
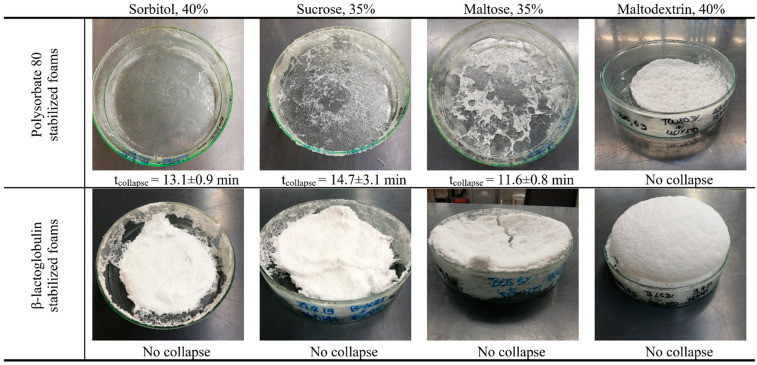
Impact of sugar types on the foam decay in conventional vacuum drying with regard to the used foaming agents and saccharides.

In Figure 6, the structure of the vacuum-dried β-lactoglobulin and polysorbate 80 stabilized foams are shown. It can be clearly observed that most PS80 stabilized foams collapsed during the first 15 min of the drying process. Only samples with maltodextrin remained stable until the end of the process. In contrast, protein samples remained stable independently of the used sugar. Here, maltose and maltodextrin showed the best results as their foam structure was preserved the best. The foams with added sucrose or sorbitol showed better foam structure but were more compact than maltose and maltodextrin. However, it was shown that the combination of surfactant and additives results in different foam stabilities, best observable for polysorbate stabilized foams.

No apparent influence of A_2_ on the drying behavior was observed for polysorbate, as the A_2_ value was similar between all investigated sugars. While all investigated sugars improved the surface properties of polysorbate due to H-bonds, the surface was getting less elastic with increasing saccharide concentration. However, interactions between polysorbate and maltodextrin, as suggested in the literature [28,54] might be the reason for the high viscosity of the solution and moreover, for the prevention of foam decay during the drying process. Those interactions were only found for MDX and not for the other investigated saccharides. Furthermore, polysaccharides were able to block the lamellae at high concentrations by their high water binding capacity and steric stabilization [53], preventing drainage and consequently foam decay, while disaccharides and sorbitol seemed not to be able to do so. However, all samples stabilized with proteins remained stable, indicating that the sugar type was not the only reason for the decay. Therefore, complex interactions between the foaming agent and the saccharide were assumed, keeping the water inside the lamellae before drainage occurs, so that the interface remains stable until solidification.

In contrast, β-lg samples showed for all in this study investigated property an improvement with the addition of sugar. On a molecular level, the charges got shielded, reducing the A_2_, and promoting the interactions between attached protein molecules. This resulted in the high elastic behavior of the interface, consequently leading to stabilization of the foam during the drying process. In contrast, higher surface tension in equilibrium did not result in worse preservation of foam. This result was also obtained for the surface activity, which was low compared to that of surfactant mixtures. One explanation for this might be that the interface was already formed, and therefore, high concentrations of foaming agents were already present at the air–water interface. Thereby, the differences between protein and surfactant samples would be lower, as the velocity of surface attachment would be negligible. In summary, all investigated combinations of β-lg and saccharide were dried successfully.

**Figure 7:**
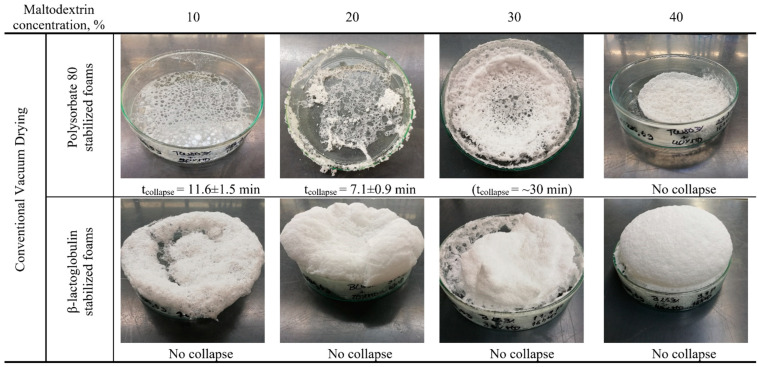
Foam structure after conventional vacuum drying for polysorbate 80 and β-lactoglobulin foam with a maltodextrin concentration between 10% and 40%.

As samples containing maltodextrin remained the most stable for both foaming agent systems, those were selected for assessing the influence of sugar concentration on foam decay during drying. As observable in Figure 7, the addition of MDX with 30% and 40% content resulted in a stable product for PS80 foams, whereas foams with 10% and 20% MDX content collapsed. One reason for this might be the higher viscosity of high carbohydrate samples. The higher viscosity of the liquid phase improves the stability of foams, as proven in several studies about foam properties [12,13,63,64]. Therefore, it is evident that a foam structure with a high viscosity is more easily stabilized against decay when compared to foams with a low viscosity. However, as β-lg samples remained stable and assuming that the viscosity between PS80 samples and β-lg samples did not differ greatly, this is less likely to be the explanation for the observed differences. Here, it was interestingly found that the tan (φ) of maltodextrin samples correlated with the result of a successful drying process as observable for PS80-MDX mixtures between 20% and 40%. This was attributed to the interactions between maltodextrin and polysorbate, promoting a stabilization of the interface while it was mechanically treated (Section 3.2). Therefore, the obtained results from samples with different polysaccharide content support this explanation. Furthermore, this would also explain why the other investigated saccharides were not able to prevent foam decay during the drying process since, for the other saccharides, the tan (φ) increased with increasing sugar content. However, regarding the tan (φ) and the saccharide concentration, two contrasting effects were observed for PS80: on the one hand, the higher the saccharide concentration, the higher tan (φ), which indicates a less elastic surface. On the other hand, the higher the saccharide concentration, the higher the viscosity of the solution, lowering the drainage speed and stabilizing the foam. Therefore, it is assumed that each mixture of saccharide and foaming agent has an optimum, resulting in overall best foam preservation properties during the drying process. Furthermore, the specific interactions between foaming agent and thickener would result in a unique foam structure for each formulation in the end of the drying process.

Besides the investigation of samples in CVD, microwave-assisted vacuum drying was performed. Differences between CVD and MWVD were assumed because components added to stabilize organic material or to increase foam stability by increased viscosity could affect MWVD due to their dielectric properties, whereas VD would only be affected by the molecular interfacial behavior of foam stabilizing substances. The obtained water activity and residual moisture content of microwave-processed samples were slightly higher compared to that of CVD samples (10–12% instead of 8–10%). However, it should be mentioned that no drying-kinetic was assessed for the CVD samples, and therefore, the results cannot be directly compared. However, the potential in decreasing drying time due to the use of microwaves for heating could be clearly demonstrated. Furthermore, it was assumed that due to higher evaporation rates during MWVD, the bubbles are better separated from each other [11] and that the formation of new bubbles and finer pores are promoted [65], whereby the aerated structure would be stabilized against foam decay. However, the influence of these mechanisms on the overall foam preservation is dependent on the process conditions, drying stage, and product formulation. Therefore, it cannot be stated which one dominates on the foam preservation during the drying process.

**Figure 8:**
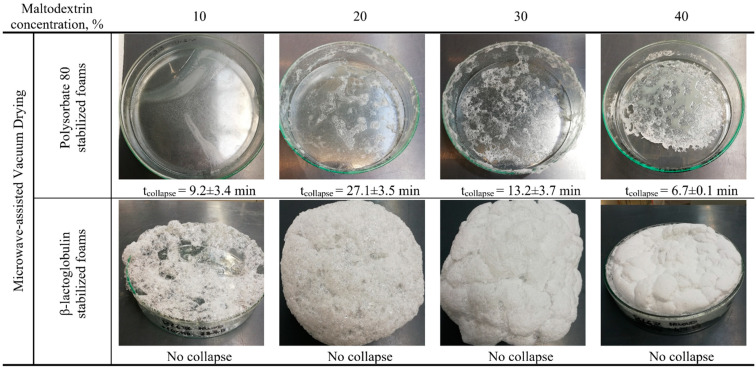
Obtained foam structure after microwave-assisted vacuum drying of polysorbate 80 and β-lactoglobulin foams with a maltodextrin content between 10% and 40%.

In Figure 8, the obtained structures for MDX concentration between 10% and 40% for both foaming systems are shown. The obtained results were clearly different compared to those of CVD: for PS80 stabilized systems, no foam remained stable during drying. The reason for this might be the too low surface elasticity of PS80 samples, as well as the dielectric properties of the solution as suggested in a previous study [66]. Herein, it was stated that the used microwave frequency matched with the resonance frequency of the foaming agent, which resulted in a decay of the foam. As it was found in this work that the elasticity of the surface was increasing with high saccharide concentration, the evidence here supports this theory. Besides, the re-formation of foam due to high evaporation rates during MWVD did not result in better preservation of the aerated structure in comparison to samples dried with VD (Figure 7). In contrast to Sankat and Castaigne [65], the samples were dried using a vacuum, which resulted in less thermal stress but higher mechanical stress. Thereby, the bubbles collapsed faster than new foam was generated due to the evaporation resulting in an overall foam collapse.

β-lg preserved the foam structure much better. However, the size of the obtained solidified bubbles was bigger, and the structure was coarser compared to the results of CVD. This might be due to the volumetric heating and faster drying. Thereby, the bubbles might have expanded more compared to CVD dryings before they solidified. Nonetheless, even for these harsh conditions, β-lg samples remained stable throughout the drying process. This was also proven for the other sugar-β-lg matrices (data not shown).

## 4. Conclusions

The obtained data demonstrate the importance of gaining knowledge about the solutions’ properties to understand the foam decay behavior during vacuum and microwave vacuum drying. Significant differences between surfactant and proteins systems were found as well as between the impact of different types of saccharides on foam stability. Furthermore, the obtained data indicated that all investigated saccharides promoted H-bonds and, therefore, indirectly or directly influenced the interactions between surface-active components. A decrease of repulsion between proteins was observed, which was correlated to the shielding effect by the added saccharides. However, the interactions within the different saccharide-foaming agent systems differed strongly, and therefore, general assumptions regarding the success of foam drying are difficult to make.

On a molecular level, the addition of saccharides had nearly no influence on the interactions between surfactants. However, if the surfactant forms complexes with the used saccharide, this statement loses its ground, as observed for maltodextrin. If it is assumed that those complexes are predominantly formed by polymers and not by small sugar alcohols or disaccharides, this effect would not be expected for most of the added excipients in the pharmaceutical industry. However, the addition of polysaccharides as thickening agents is quite common in the food industry and should be considered for other areas, too.

The addition of saccharides to β-lactoglobulin solutions improved the surface properties of the system. Repulsion between β-lg molecules was lowered, and the intermolecular interactions at the interface were promoted. Thereby, the surface tension was slightly lowered. However, no correlation between surface tension, surface activity, and foam decay during drying was found. In contrast, the obtained results of surface rheology were well correlated with the obtained drying results. All β-lg samples showed high elastic behavior at the interface and preserved the foam structure during the drying. Nevertheless, polysorbate samples were also showing high elastic surfaces (low tan (φ)) for some saccharide concentrations, and therefore the tan (φ) cannot be used as an overall explanation. However, a good prediction of foam decay during the drying process can be made using a combination of E’ and tan (φ). Therefore, it is recommended to test formulations, which should be vacuum foam dried, for their surface rheological properties.

The obtained microwave processed samples always showed more indications for foam decay compared to CVD samples. This was attributed to the harsher drying conditions as a result of faster evaporation and more efficient heating. However, β-lactoglobulin stabilized samples were robust enough to withstand those conditions. Investigations about the dielectric properties of the solutions might also be relevant to go deeper into the drying technology and the optimization of drying parameters, such as microwave power input, vacuum pressure, and drying temperature. With regard to the foam preservation, a formulation of 3% β-lg and 40% MD was best suited.

Overall, a correlation between molecular and interfacial properties and the drying results in terms of maintaining stable foams was established. This could be used for an upfront characterization of solutions to predict the drying behavior. Furthermore, it can be estimated that samples using whey protein isolate instead of β-lg result in comparable product characteristics as shown in a previous study [67].

## Figures and Tables

**Table 1:** Second virial coefficient A_2_ of polysorbate 80 and β-lactoglobulin solutions in the appearance of different saccharides at 20 °C

Foaming Agent	Saccharide	A_2_ (10^−7^, mol·dm^3^·g^−2^)
Polysorbate 80	Sorbitol	1.38 ± 0.36 ^a^
	Sucrose	1.08 ± 0.33 ^a^
	Maltose	1.18 ± 0.22 ^a^
	Maltodextrin	1.20 ± 0.20 ^a^
β-lactoglobulin	Sorbitol	11.30 ± 2.61 ^b^
	Sucrose	14.17 ± 7.86 ^b^
	Maltose	6.80 ± 1.73 ^b^
	Maltodextrin	9.58 ± 3.39 ^b^

^a,b^ Means in the same column with different letters indicate significant differences (*p* < 0.05).

**Table 2:** Calculated surface activity of samples with polysorbate 80 and β-lactoglobulin in the presence of added saccharides with content between 0% and 30% at 20 °C

	β-Lactoglobulin, mN·m^−1^·s^−1^				Polysorbate 80, mN·m^−1^·s^−1^			
c (Saccharide, %)	Sorbitol	Sucrose	Maltose	Maltodextrin	Sorbitol	Sucrose	Maltose	Maltodextrin
0	−1.62 ± 0.35 ^a^	−1.62 ± 0.35 ^a^	−1.62 ± 0.35 ^a^	−1.62 ± 0.35 ^a^	−12.53 ± 6.44 ^a^	−12.53 ± 6.44 ^a^	−12.53 ± 6.44 ^a^	−12.53 ± 6.44 ^a^
10	−1.60 ± 0.26 ^a^	−1.79 ± 0.31 ^a^	−1.51 ± 0.34 ^a^	−1.40 ± 0.30 ^a^	−12.72 ± 6.55 ^a^	−13.02 ± 7.01 ^a^	−14.63 ± 7.75 ^a^	−4.40 ± 1.80 ^a,c^
20	−1.56 ± 0.29 ^a^	−2.26 ± 0.46 ^a^	−1.32 ± 0.19 ^a^	−2.38 ± 0.68 ^a^	−13.18 ± 6.81 ^a^	−14.29 ± 7.33 ^a^	−14.95 ± 8.06 ^a^	−1.07 ± 0.12 ^b^
30	−1.18 ± 0.16 ^a^	−2.33 ± 0.40 ^a^	−1.37 ± 0.11 ^a^	−2.46 ± 0.56 ^a^	−2.35 ± 0.82 ^a^	−4.82 ± 2.05 ^a^	−5.00 ± 2.21 ^a^	−1.67 ± 0.38 ^b,c^

^a–c^ Means in the same column with different letters indicate significant differences (*p* < 0.05).

**Table 3:** Calculated tan (φ), -for samples with polysorbate 80 or β-lactoglobulin at a saccharide concentration of 20% at 20 °C

	Foaming Agent	Maltodextrin	Maltose	Sucrose	Sorbitol
Polysorbate 80	0.21 ± 0.03 ^a^	0.54 ± 0.02 ^b^	0.28 ± 0.02 ^c^	0.22 ± 0.02 ^a^	0.31 ± 0.01 ^c,d^
β-lactoglobulin	0.23 ± 0.01 ^a^	0.18 ± 0.01 ^b^	0.22 ± 0.01 ^a,c^	0.25 ± 0.02 ^a,c^	0.21 ± 0.01 ^a,c^

^a–d^ Means in the same line with different letters indicate significant differences (*p* < 0.05).

## Data Availability

Data is contained within the article.
